# Editorial: Animal-friendly methods for rodent behavioral testing in neuroscience research

**DOI:** 10.3389/fnbeh.2024.1431310

**Published:** 2024-06-25

**Authors:** Raffaele d'Isa, Stefania Fasano, Riccardo Brambilla

**Affiliations:** ^1^Institute of Experimental Neurology (INSPE), Division of Neuroscience (DNS), IRCCS San Raffaele Scientific Institute, Milan, Italy; ^2^Neuroscience and Mental Health Innovation Institute, School of Biosciences, Cardiff University, Cardiff, United Kingdom; ^3^Department of Biology and Biotechnology “Lazzaro Spallanzani”, University of Pavia, Pavia, Italy

**Keywords:** behavioral testing, rodents, animal welfare, refinement, animal-friendly testing

## Background

Rodents have been employed in modern scientific research from the 17th century. However, in the early period of experimental science, scientists often had scarce attention for animal welfare. For instance, in 1659 Irish-English chemist Robert Boyle (1627–1691) performed suffocation tests on mice placed in extremely rarefied air and measured the time the animals took to die without air to breathe (Boyle, 1660). On the other hand, Italian biologist Francesco Redi (1626–1697) performed terminal starvation tests on both domestic mice and field mice to discover how much time they could survive without food, finding that both species were dead within 3 days (Redi, 1684). In the following century, rodents were employed mainly in lethal experiments, for example the toxicological studies of the Italian naturalist Felice Fontana (1730–1805), director of the Museum of Natural History of Florence from 1775, who used guinea pigs to assess the effects of inflammable air (Fontana, 1779), curare venom (Fontana, 1780, 1781, 1787), cherry-laurel poison (Fontana, 1781, 1787) and viper venom (Fontana, 1781, 1787). While toxicological studies were driven by a practical utility, other studies of that time, by inflicting pointless suffering, appear as merely cruel.

Compared to other non-human animals such as dogs and cats, it has been relatively more difficult for humans to empathize and sympathize with rodents such as mice and rats, probably also because they have often been viewed as pest animals infesting urban environments or damaging orchards, agricultural cultivations and cereal deposits (Stenseth et al., 2003), and because they were perceived as “lower” animals. Indeed, the idea that rodents are cognitively inferior animals could be one of the reasons for which the welfare of laboratory rodents has often been overlooked, especially in the past centuries of scientific research. Importantly, the cognitive limitedness of rodents has been challenged by the neuroscientific and psychological investigations of the past few decades, which have revealed increasingly complex cognitive, emotional and social skills for these animals (Langford et al., 2006; Miller, 2006; Rutte and Taborsky, 2007, 2008; Viana et al., 2010; Ben-Ami Bartal et al., 2011; Dolivo et al., 2016; Zentall, 2016; Schweinfurth and Taborsky, 2018a,b; Sivaselvachandran et al., 2018; Ueno et al., 2018; Mogil, 2019; Reinhold et al., 2019; Templer, 2019; Cox and Reichel, 2020; Venniro and Golden, 2020; Joo et al., 2021; Kim et al., 2021; Rutishauser, 2021; Hernandez-Lallement et al., 2022; Engelhardt and Taborsky, 2023; Keysers and Gazzola, 2023; Misiołek et al., 2023; Yu et al., 2024).

However, it is important to underline that the criterion for the right for animal welfare should not be the cognitive level of a species, but rather its ability to feel. In the words of the British philosopher Jeremy Bentham (1748–1832), one of the first to criticize specist prejudices and the adoption of intelligence as criterion to decide whether a given species deserves welfare concerns: “The French have already discovered that the blackness of skin is no reason why a human being should be abandoned without redress to the caprice of a tormentor. It may come one day to be recognized, that the number of legs, the villosity of the skin, or the termination of the os sacrum, are reasons equally insufficient for abandoning a sensitive being to the same fate. What else is it that should trace the insuperable line? Is it the faculty of reason, or perhaps, the faculty for discourse? [...] the question is not, Can they reason? nor, Can they talk? but, Can they suffer?” (Bentham, 1789). Actually, as argued by philosopher Sahar Akhtar in *The Oxford Handbook on Ethics and Animals*, for non-human animals pain may be even worse than for humans, as non-human animals lack the possibility to rationalize about the causes of their pain, or to imagine a future in which the pain has ceased (Akhtar, 2011). As eloquently expressed by the American bioethicist Bernard E. Rollin (1943–2021): “If they are in pain, their whole universe is pain; there is no horizon; they are their pain” (Rollin, 1999).

Behavioral studies on rodents kept in a controlled environment (i.e., not in nature) began to be carried out much later than physiological studies. Indeed, behavioral studies on captive rodents were first performed in 1822 (Moss, 1836) and became more common from the 1870s (Jillson, 1871; Lockwood, 1871; Tenney, 1872; Perkins, 1873; King, 1883; Stephens, 1887; Davis, 1889). Nevertheless, these first behavioral studies were merely observational. Behavioral testing of rodents started instead in the 1890s (Stewart, 1894, 1898; Lombard, 1895; Mills, 1895, 1898; Kline, 1899a,b; Small, 1899).

Rodent behavioral testing has since then been employed in an increasingly growing number of studies to investigate brain functions, and has become a gold-standard method in modern neuroscience. With the study of behavior, came also a greater attention for the welfare of laboratory rodents, both because their cognitive abilities were better understood and because of the awareness that affecting the welfare of the animals could impact the scientific results of behavioral studies. As noted by Small, one of the pioneers of rodent behavioral testing: “the experiments must conform to the psycho-biological character of an animal if sane results are to be obtained” (Small, 1901). Nevertheless, the first behavioral methods developed for laboratory rodents often still had a great margin of improvement for optimization of animal welfare.

Indeed, the vast majority of rodent behavioral tests designed up to the 1950s was based on punishments and rewards. Unfortunately, both these approaches can lead to a certain degree of animal pain or suffering. Punishments required the employment of painful stimuli, typically electric shocks. Tests as passive avoidance and fear conditioning can be performed using only a single brief shock, but other tests, as active avoidance, can require tens or even hundreds of shocks, which make them an extreme challenge for the psychological welfare of the animals. On the other hand, tests based on rewards, which apparently may seem more ethical, actually still induce suffering in the animals, as food rewards are almost always associated with a food restriction protocol, in order to motivate the animals to seek food. In this case, the rodents are starved for days before starting the test and kept under food restriction for the whole duration of the test. For the radial maze, for example, animals will suffer hunger for 2 weeks (3–4 days of pre-training phase and 10 days of training). Actually, the distress during the testing session is only a minimal part compared to the stress lived outside of the testing session, which is prolonged and continuous. Analogously, liquid rewards commonly rely on a previous water restriction protocol, in order to use thirst as motivation for reward-seeking.

**Figure 1:**
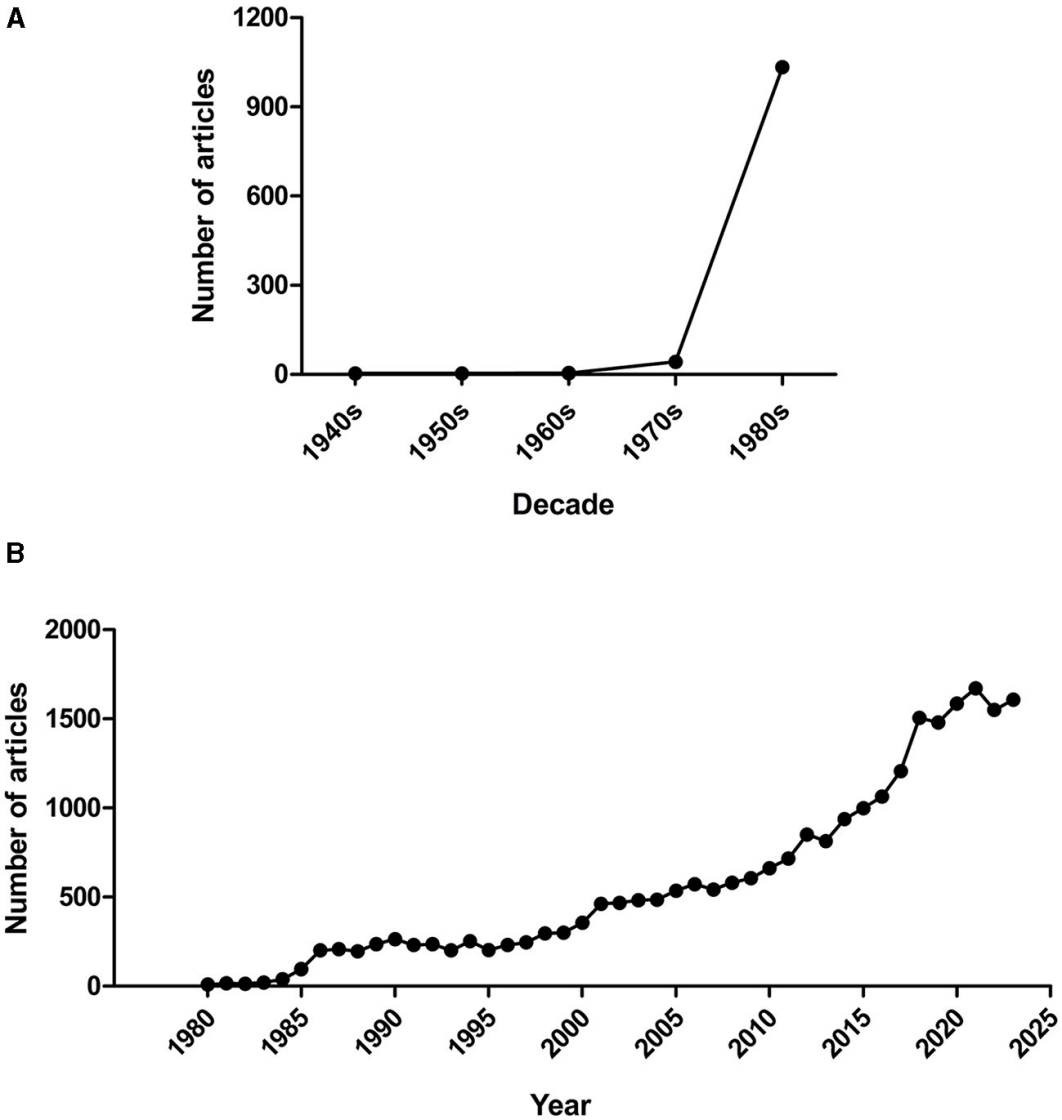
Number of publications mentioning “animal welfare”. **(A)** Number of articles, listed in the PubMed database, mentioning the phrase “animal welfare”, per decade (from the 1940s, when the first article employed the phrase, to the 1980s). **(B)** Number of articles, listed in the PubMed database, mentioning the phrase “animal welfare”, per year (from 1980 to 2023).

Animal stress is not only an ethical issue *per se*, but is also an important factor that puts at risk the reliability and reproducibility of scientific results. From the 1960s, many tests have been designed that do not employ punishments or rewards, being based on spontaneous behaviors of the rodents. For instance, in Boissier and Simon's 16-hole-board (Boissier and Simon, 1962) or File and Wardill's 4-hole-board (File and Wardill, 1975; d'Isa et al., 2021a), mice are induced to look inside the holes of a board simply by their natural curiosity. In Ennaceur and Delacour's object recognition test mice are exposed to objects, which are spontaneously explored on the basis of their novelty (Ennaceur and Delacour, 1988; d'Isa et al., 2014). In the object location test, the displacement of an object is used to create a source of novelty and hence induce higher levels of exploration (Ennaceur et al., 1997). Maze examples are the spontaneous alternation T-maze (d'Isa et al., 2021b) and the continuous alternation Y-maze (Gerlai, 1998). The attention of the biomedical community for animal welfare sharply increased over the course of the past 40 years. To provide a metric, a search in the PubMed biomedical archive shows that the number of new scientific articles mentioning the phrase “animal welfare” remained constantly under 50 for each decade from the 1940s (when the first article with “animal welfare” was published) to the 1970s, but underwent an explosion in the 1980s, reaching more than 1000 hits (Figure 1A). From the 1980s, the annual number of new articles progressively increased up to present, indicating an escalating growth, and got to more than 1600 in 2023 alone (Figure 1B).

In May 2022, with the present Research Topic, we launched a call to encourage works on animal-friendly behavioral testing methods for rodents. The call was received with enthusiasm by the scientific community. Indeed, the article collection that we are glad to present here comprises 20 contributions by 70 authors from countries across the world, ranging from Norway to Mexico and from California to Japan. Several different approaches have been explored, from automated home-cage monitoring to robotic rats, and from seminatural environments to freely-accessible mazes directly connected to the home-cage. We will hereon briefly describe the Research Topic contributions.

## General concepts

Two articles of the Research Topic deal with general concepts related to animal-friendly testing. In the first, d'Isa and Gerlai underline the importance of the employment of knowledge of the species-specific peculiarities of a given species to design behavioral tests that produce valid results (not random one-time responses to an artificial situation) and that are based on animal-friendly motivators. This combined maximization of ethological validity and animal welfare is a key feature of what the authors defined as ethological neuroscience. Additionally, the authors present a rating scale for behavioral tests, which is based on their impact on animal welfare and features 12 levels, from A (animal-friendly) to L (lethal). It is the hope of the authors that in future an increasing number of A-level behavioral tests will be designed.

Comparative psychologist Charles I. Abramson has investigated, over the course of almost half a century, the behavior of more than 40 species. In the present Research Topic, Abramson explains the importance of comparative psychology for neuroscientists. Indeed, according to Abramson, the comparative approach can be a valuable *forma mentis* for the study of behavior. For instance, the analysis of the behavioral differences of closely related species, or even strains, can help to elucidate, by subtraction, the genetic and neural underpinnings of behavior, and it can allow to identify more easily species-specific peculiarities that can be useful for the design of species-tailored behavioral tests that optimize animal welfare.

In the following paragraphs we will describe the contributions dealing with specific behavioral tests, subdivided into two categories: closed-session and open-session behavioral tests. In the first, the subject animals are brought to a specific testing environment different from their living environment, they are tested at a specific hour of the day chosen by the experimenter, the duration is a fixed short period (generally between 1 and 60 min) and the animals are returned to their living environment only at the conclusion of the testing session. On the other hand, in open-session behavioral tests, the animals: (1) remain in their living environment; (2) are given the opportunity to approach freely a series of interactive testing elements; (3) can choose the moment of the day when they want to start behavioral testing and for how long to engage in the testing; (4) undergo open-session testing, meaning that they can stop and resume behavioral testing in any moment, alternating testing with their regular living activities, such as feeding or sleeping.

## Closed-session animal-friendly behavioral tests

Among closed-session tests, a good example of animal-friendly behavioral testing is the paced mating test, standardized by Mary Erskine (1946–2007) in the 1980s (Erskine, 1985, 1987; Erskine et al., 1989) and made more widely popular by Raúl Paredes and collaborators between the late 1990s and the early 2000s (Paredes and Alonso, 1997; Paredes and Vazquez, 1999; Martinez and Paredes, 2001; Paredes and Martinez, 2001). In the present Research Topic, Ventura-Aquino and Paredes describe the usefulness of this test to investigate behavioral, neuroendocrine and neuroplastic changes in female rats and mice following sexual experience. In traditional non-paced mating tests, the females are exposed to a sexually active male and cannot escape from its approaches. In such a situation, sexual activity may lose its rewarding value and become stressful for the females. In contrast, in nature, female rats and mice have the possibility to accept or reject sexual approaches from the male, a possibility which prompts male courtship efforts and is at the basis of biological evolution through sexual selection. Paced mating reproduces in laboratory the same possibility, enabling the females to choose if, when and for how long engage in sexual activities with the males. Such protocol, on the one hand, increases the ethological validity of the behavioral test and, on the other hand, it increases the animal welfare of the experimental subjects.

Comparative psychologist Shigeru Watanabe, who has been professor at Keio University in Tokyo for over 40 years, contributed to the Research Topic with two works [Watanabe (a, b)]. In the first, he proposes the possibility to employ mirror-based tests which use the mirror as an animal-friendly reward not requiring previous food or water deprivation [Watanabe (a)]. In the second, Watanabe reviews the use of a non-invasive and contactless technique, infrared thermography, to evaluate social judgements in mice and highlights the potential of this non-invasive tool for the animal-friendly study of cognition and emotion in rodents [Watanabe (b)]. This approach has currently been employed in various rodents, including laboratory mice (Watanabe, 2015), laboratory rats (Wongsaengchan et al., 2023) and wild mice (Delacoux and Guenther, 2023), as well as in many non-rodent species (Mota-Rojas et al., 2021).

Behavioral neuroscientist Sergio Pellis, who has been investigating play behavior for over 45 years, and colleagues propose the rough-and-tumble play of juvenile rats as a natural behavior offering a unique window to study the processes of the social brain (Pellis et al.). Indeed, the rough-and-tumble play is a playful confrontation, highly pleasurable for the participants, in which competition for physical dominance is moderated by cooperation, including self-limitation and turn taking, which leads to a voluntary exchange of the dominant and submissive roles. This complex play behavior is particularly suitable to test social decision-making in rats.

In social interaction tests featuring encounters between unfamiliar adult rodents, a subject animal is exposed to an unfamiliar stimulus animal, and the behavior of the subject animal is scored. However, such encounters have the problem that, especially with males, fights may often occur, with the possibility of pain and injuries for the animals involved. In specific paradigms as the resident-intruder test, the risk of fight-related injuries is even higher (Koolhaas et al., 2013). Different solutions can be imagined to solve this issue. Harda et al. performed a partitioned social interaction test, in which the two animals were separated by a transparent perforated barrier that allowed the mice to see and smell, but not touch, each other, as well as a second test with the stimulus mouse placed inside a protective wire-mesh cup in an open-field arena. Through these tests, the authors showed that C57BL/6N mice have a sub-strain specific resistance to ketamine-induced social behavior deficits. Robotics engineer Siddall proposes a solution that additionally allows physical interaction: the employment of robotic animals as stimulus animals. In particular, Siddall reports the characteristics of 13 models of robotic rats that have been developed over the course of the past 20 years, and describes which features the robotic rats of the future should possess to be employed effectively in behavioral research. The use of robotic rats would not only make social interaction tests safe, but it would also, since the behavior of the robotic rats is programmable or remotely controllable by the experimenters, allow an unprecedented control over the experimental design.

The pup retrieval test is currently the leading procedure to assess maternal behavior in rodents. Winters et al. present an automated version of the test that, for the first time, allows synchronous video-recording of maternal behavior and audio-recording of pup vocalizations, which allows to assess bidirectionally the dam-pup dyadic interaction. This new test, named BAMBI (Bidirectional Automated Mother-pup Behavioral Interaction), is performed in the home-cage and employs artificial intelligence for computer vision allowing body part tracking and pose estimation, as well as for automated audio-recognition of pup ultrasonic calls.

Finally, Nunes, who has been studying squirrels for over 35 years, argues how animal-friendly rodent behavioral tests can be used also in the field. In particular, Nunes describes animal-friendly behavioral tests that can be performed *in situ* on free-living ground squirrels, including tests for motor coordination, the caution-boldness continuum, docility and problem-solving.

## Open-session animal-friendly behavioral tests

The best example of open-session animal-friendly behavioral testing are the automated home-cage monitoring systems (Mingrone et al., 2020; Voikar and Gaburro, 2020; Grieco et al., 2021; Kahnau et al., 2023), which avoid potentially stressful animal handling, do not require removal of the animals from their home-cage for testing in unfamiliar and hence potentially anxiogenic environments, permit the animals to be tested in a social context together with their mates and respect the circadian rhythms of the tested subjects. The most widely known of these systems is IntelliCage, conceived by Hans-Peter Lipp and collaborators in the early 2000s at the University of Zurich (Galsworthy et al., 2005; Lipp, 2005; Lipp et al., 2005). In this smart cage, the interactions of mice or rats with specific elements (visits to the corners, nose-pokes to nose-holes and licks of the bottle-nipples) are recorded automatically and continuously, allowing to design different experimental protocols for the evaluation of motor and cognitive functions. In our Research Topic, Lipp, who has been studying animal behavior for 50 years, traces with colleagues the history of the development and the future perspectives of this animal- and user-friendly automated behavioral testing system (Lipp et al.). The authors also present an evolution of IntelliCage: greater-sized chambers endowed with the same interactive elements of the smart home-cages. These IntelliCage-like environments, which could be named IntelliChambers, have already been tested with marmosets and could be particularly useful for the behavioral testing of rodents that require abundant space to move along three dimensions, such as squirrels and chinchillas.

Four experimental works of our Research Topic employed IntelliCage. Many standard learning protocols in IntelliCage use controlled water access as the motivational driver. However, this may lead to water restriction in slow learners. Bramati et al. present a new IntelliCage learning protocol in which mice have permanent access to plain water but can additionally be rewarded with saccharin-sweetened water if during the task they perform a correct choice. Through this appetitively motivated learning protocol, the authors showed that environmental enrichment enhances hippocampus-dependent spatial learning in female mice (Bramati et al.). Nevertheless, while this purely appetitive motivator was effective for simple tasks, an excessive number of mice lost interest in the sweet reward when challenged with more difficult hippocampus-dependent tasks. To solve this issue, Ma et al. compared, in female mice, the purely appetitive task (correct choice: saccharin; wrong choice: water) with other two new tasks, in which the second option (water) was devalued by (a) the addition of bitter-tasting quinine, or (b) increasing the number of work (nose-pokes) required to obtain it. Compared to the previous protocol (saccharin vs. water), these two novel combined incentive-disincentive protocols showed a strong improvement of both task engagement and task performance. Nigri et al. tested the Bramati protocol in male mice, finding that for the males the performance levels dropped even more rapidly than for the females when switching from simple to complex learning tasks, suggesting a higher motivational cost for the males. New protocols optimizing the performance of males are yet to be tested, but a suggestion could come from the combined incentive-disincentive protocols conceived by Ma et al.. Finally, Wu et al. employed a water-motivated IntelliCage protocol in which access to water could be denied only for a maximum of 2.5 h, in order to avoid dehydration and psychological stress derived from thirst. Through this protocol, the authors found that stimulation or inhibition of GABA_B_ receptors in the insula of epileptic rats led to, respectively, reduced or increased memory, in both spatial and non-spatial operant tasks.

Another home-cage behavioral monitoring system is the Home Cage Analyser (HCA; Bains et al., 2016). Here, Bains et al. present a new method for HCA based on a computer vision algorithm capable of measuring climbing on the wire lid of the home-cage. Home-cage monitoring of climbing behavior allowed early detection (at 8 weeks) of motor impairment in the N171-82Q mutant mouse, a widely employed model of Huntington's disease, suggesting an interesting new behavioral marker for this neurological disease. Additionally, in healthy mice, a sex effect was found, with females spending more time climbing than males.

Julius Emmrich's team at the German Center for the Protection of Laboratory Animals has recently developed a new refined version of the radial maze which is fully automated, handling-free, voluntary and does not require food or water deprivation (Mei et al., 2020). In this test, the maze is connected through a tube to the home-cage, and the mice can freely decide when to explore the maze and perform the spatial memory testing. In the present Research Topic, the same team perfected the method and directly compared the refined radial maze with the classical radial maze (Kohler et al.). Both tests showed significant learning in healthy mice and detected spatial impairments in lipopolysaccharide (LPS)-injected mice.

Hernández-Arteaga and Ågmo describe the benefits of employing seminatural environments for rodent behavioral testing. These settings, which reproduce in the laboratory an environment similar to the natural one, are particularly appropriate for the study of sexual behavior. Indeed, in seminatural environments, as in nature, males and females equally control the sexual interactions (Bergheim et al., 2015). As in closed-session paced mating, in seminatural environments male sexual approaches are escapable by females. Moreover, females perform proceptive behaviors that incite male copulation and that can be considered as an index of female sexual motivation. Importantly, Bergheim and colleagues found that, in a seminatural environment, the almost totality of copulatory acts (96%) were performed within 5 s from a female proceptive behavior, indicating a high level of sexual motivation in the females. Additionally, sexual interactions were initiated by females as frequently as by males. Overall, seminatural environments not only are research tools more suitable for the animal welfare of the female subjects, but additionally they represent a more realistic and ethologically valid model of bidirectional socio-sexual interactions between males and females.

Finally, Parsons et al. outline the advantages of the free exploratory paradigm (FEP), which can be used both in the laboratory (as in Kohler et al.) and in the wild (Parsons et al., 2023). Indeed, by placing free-access test chambers in natural environments, rodent behavior can be assessed without handling, relying on spontaneous activity, avoiding the need of keeping animals in captivity and in a context with a higher ecological validity. Moreover, non-conventional species of rodents, such as field mice, can be studied and heterozygosity-enriched groups could be employed.

## Conclusions

The present Research Topic includes numerous different approaches for animal-friendly behavioral testing. In future, hopefully, each of these approaches will be further developed and new approaches will be found. However, the most interesting frontier of the evolution of animal-friendly behavioral testing could be, rather than the amelioration of a single approach, the combination of different approaches. For instance, robotic rats could be placed in seminatural environments for rat-robot social interactions. IntelliChambers could host seminatural environments, as well as robotic rats. And so on. Since several new approaches and technologies have become available, scientists will be free to use all their creativity and ingenuity to combine these options at best and design optimal paradigms for animal-friendly behavioral testing.

## Author contributions

RdI: Conceptualization, Project administration, Supervision, Visualization, Writing – original draft, Writing – review & editing. SF: Conceptualization, Writing – review & editing. RB: Conceptualization, Writing – review & editing.
